# Intensive versus Guideline Blood Pressure and Lipid Lowering in Patients with Previous Stroke: Main Results from the Pilot ‘Prevention of Decline in Cognition after Stroke Trial’ (PODCAST) Randomised Controlled Trial

**DOI:** 10.1371/journal.pone.0164608

**Published:** 2017-01-17

**Authors:** Philip M. Bath, Polly Scutt, Daniel J. Blackburn, Sandeep Ankolekar, Kailash Krishnan, Clive Ballard, Alistair Burns, Jonathan Mant, Peter Passmore, Stuart Pocock, John Reckless, Nikola Sprigg, Rob Stewart, Joanna M. Wardlaw, Gary A. Ford, PODCAST Trial Investigators

**Affiliations:** 1 Stroke Trials Unit, Division of Clinical Neuroscience, University of Nottingham, Nottingham, United Kingdom; 2 Sheffield Institute for Translational Neuroscience, University of Sheffield, Sheffield, United Kingdom; 3 Department of Neurology, Sandwell and West Birmingham Hospitals NHS Trust, Birmingham, United Kingdom; 4 Wolfson Centre for Age-Related Diseases, King’s College London, Guy’s Campus, London, United Kingdom; 5 Faculty of Medical and Human Sciences, Institute of Brain, Behaviour and Mental Health, University of Manchester, Manchester, United Kingdom; 6 General Practice & Primary Care Research Unit, University of Cambridge, Addenbrooke’s Hospital, Cambridge, United Kingdom; 7 Institute of Clinical Sciences, Queens University, Belfast, Royal Victoria Hospital, Belfast, United Kingdom; 8 Department of Medical Statistics, London School of Hygiene and Tropical Medicine, London, United Kingdom; 9 Department of Endocrinology, Royal United Hospital, Bath, United Kingdom; 10 Department of Psychological Medicine, King's College London (Institute of Psychiatry, Psychology and Neuroscience), London, United Kingdom; 11 Centre for Clinical Brain Sciences, Western General Hospital, Edinburgh, United Kingdom; 12 Medical Sciences Division, University of Oxford, John Radcliffe Hospital, Oxford, United Kingdom; University of Glasgow, UNITED KINGDOM

## Abstract

**Background:**

Stroke is associated with the development of cognitive impairment and dementia. We assessed the effect of intensive blood pressure (BP) and/or lipid lowering on cognitive outcomes in patients with recent stroke in a pilot trial.

**Methods:**

In a multicentre, partial-factorial trial, patients with recent stroke, absence of dementia, and systolic BP (SBP) 125–170 mmHg were assigned randomly to at least 6 months of intensive (target SBP <125 mmHg) or guideline (target SBP <140 mmHg) BP lowering. The subset of patients with ischaemic stroke and total cholesterol 3.0–8.0 mmol/l were also assigned randomly to intensive (target LDL-cholesterol <1.3 mmol/l) or guideline (target LDL-c <3.0 mmol/l) lipid lowering. The primary outcome was the Addenbrooke’s Cognitive Examination-Revised (ACE-R).

**Results:**

We enrolled 83 patients, mean age 74.0 (6.8) years, and median 4.5 months after stroke. The median follow-up was 24 months (range 1–48). Mean BP was significantly reduced with intensive compared to guideline treatment (difference –10·6/–5·5 mmHg; p<0·01), as was total/LDL-cholesterol with intensive lipid lowering compared to guideline (difference –0·54/–0·44 mmol/l^;^ p<0·01). The ACE-R score during treatment did not differ for either treatment comparison; mean difference for BP lowering -3.6 (95% CI -9.7 to 2.4), and lipid lowering 4.4 (95% CI -2.1 to 10.9). However, intensive lipid lowering therapy was significantly associated with improved scores for ACE-R at 6 months, trail making A, modified Rankin Scale and Euro-Qol Visual Analogue Scale. There was no difference in rates of dementia or serious adverse events for either comparison.

**Conclusion:**

In patients with recent stroke and normal cognition, intensive BP and lipid lowering were feasible and safe, but did not alter cognition over two years. The association between intensive lipid lowering and improved scores for some secondary outcomes suggests further trials are warranted.

**Trial Registration:**

ISRCTN ISRCTN85562386

## Introduction

Stroke is complicated by cognitive impairment in up to 92% of survivors,[1] and dementia in 30%. Post stroke cognitive impairment (PSCI) more commonly affects executive dysfunction, and is associated with increased mortality and decreased quality of life.[2, 3] Despite these serious complications, which are devastating to patients and their family and economically costly to society, the evidence base for the prevention of PSCI and post-stroke dementia (PSD) is limited. Many potential interventions for preventing cognitive decline have been proposed, including blood pressure (BP) and lipid lowering, antiplatelet agents, anti-oxidant vitamins, and cholinesterase inhibitors.[4] Of these, lowering BP and blood lipid levels are priorities for testing as elevated BP and cholesterol are common after stroke, effective therapies are available, and consistent trial evidence supports drug treatment to prevent recurrent vascular events.[5–7] As a result, most patients need their BP lowered, and those with ischaemic stroke usually need a statin, as recommended in guidelines.[8, 9]

Although the effect of BP lowering on cognitive impairment and dementia has been assessed in several trials, the results are conflicting and only hypothesis-generating since cognitive outcomes were never the primary outcome in these trials. In a post-stroke population, the PROGRESS trial found that perindopril, with or without indapamide (versus placebo), reduced PSCI and PSD, largely through preventing stroke recurrence.[10, 11] In contrast, both the PRoFESS (telmisartan versus placebo) and SPS3 (intensive versus guideline BP lowering in patients with subcortical stroke) trials reported no benefit of antihypertensive therapy on cognition post stroke.[12–15] A meta-analysis of these trials, and others not involving stroke patients, found that BP lowering was associated with less cognitive decline (assessed as change in the Mini-Mental State Examination, MMSE) but not with reduced dementia;[4] a meta-regression of the same studies suggested that there might be a ‘J-shaped’ curve linking the development of dementia with difference in BP between treatment groups.[4] In a meta-analysis limited to patients without stroke, BP lowering was associated with less cognitive decline but not dementia.[16]

In respect of lipid lowering, there is no positive evidence that statins (currently the main lipid intervention) prevent cognitive decline or dementia, as reported in secondary analyses from the PROSPER (pravastatin) and HPS (simvastatin) trials.[17–19] Overall, systematic reviews of trials using statins to prevent or treat dementia have reported no benefit.[4, 20, 21] However, systematic reviews of observational and other studies raised the possibility that statins might reduce dementia.[22, 23]

In view of the uncertainties surrounding the potential for BP and lipid lowering to reduce PSCI and PSD, and yet the need for most patients to receive antihypertensive and lipid lowering therapy for the purposes of secondary prevention, the pilot PODCAST randomised controlled partial-factorial trial compared intensive versus guideline BP lowering, and intensive versus guideline lipid lowering, on cognition and other functional measures, and the main results are reported here.

## Methods

PODCAST was a pilot multicentre prospective randomised open-label blinded-endpoint (PROBE) trial. Details of the design, statistical analysis plan and baseline data have been published;[24, 25] the full protocol (S2 File) is available online (http://www.podcast-trial.org/PodcastProtocolV16.pdf). In brief, patients with a recent stroke were randomly assigned to at least 6 months of management with intensive BP lowering or guideline BP lowering. In addition, the subset of patients with ischaemic stroke were randomly assigned to intensive vs guideline lipid lowering in a partial-factorial design.[24]

Patients were eligible for the trial if they had an ischaemic or haemorrhagic stroke within the previous 3 to 7 months, were aged over 70 with a telephone-Mini Mental State Examination (t-MMSE) >16 or were aged 60–70 with t-MMSE 17–20 (the latter so as to enrich the population with stroke survivors at greater risk of developing cognitive impairment and dementia), were functionally independent (modified Rankin Scale, mRS 0–2), had a systolic BP of 125–170 mmHg, had an informant who could provide information on the IQ-CODE,[26] and had capacity and were willing to give consent.[24] Patients with an ischaemic stroke also had to have a total cholesterol of 3–8 mmol/l. The diagnosis of the stroke must have been confirmed with CT or MRI using standard imaging techniques and done within 10 days of the index event to allow differentiation of stroke type. Key exclusion criteria included an existing diagnosis of dementia, need for intensive BP or lipid control, intolerance to high intensity statins, need for treatment with an acetyl-cholinesterase inhibitor, dementia, subarachnoid haemorrhage, secondary intracranial haemorrhage, insufficient capacity to provide informed consent or to complete study measures, profound deafness, chronic renal failure or eGFR <45 (or eGFR <37 in people of African/Afro-Caribbean origin), liver disease or ALT >3 times the upper limit of normal, ongoing participation in a clinical trial of an investigational medicinal product and/or device, any serious medical co-morbidity, or familial stroke associated with dementia.[24]

The study was approved by the United Kingdom (UK) National Research Ethics Committee East Midlands–Nottingham 1. Hospital local research & development offices at each site provided local approval and oversight. As a management rather than treatment trial, the study did not fall under the remit of the UK Medicines and Healthcare Products Regulatory Authority. The trial was overseen by a trial steering committee, this including two independent members and three patient & carer representatives. The day-to-day conduct of the trial was run by a trial management committee that was based at the coordinating centre in Nottingham, UK. An independent data monitoring committee reviewed unmasked data on three occasions. We obtained written informed consent from each patient before enrolment and in accordance with UK regulations. The National Institutes Health Research Stroke Research Network supported the trial through screening and recruitment of patients.[24]

### Randomisation

Investigators entered baseline and follow-up data into a database via a secure web-based randomisation system. The data were checked to confirm the patient’s eligibility in real time, and the computer system assigned participants, with allocation equally to intensive versus guideline BP lowering and, in patients with ischaemic stroke, to intensive versus guideline lipid lowering, each for a minimum of 6 months. Treatment assignment was stratified on the basis of stroke type (ischaemic *vs* haemorrhagic); and minimised on key prognostic baseline variables: age, sex, number of antihypertensive drugs, on a statin, presence of dysphasia, Addenbrooke’s Cognitive Examination-R (ACE-R), systolic BP, total cholesterol, mRS, cortical or subcortical stroke,[27] evidence of periventricular white matter changes on scan, time since index stroke; minimisation included a random element in 5% of patients. We used stratification and minimisation to ensure that the groups were balanced for prognostic factors, and the random element reduced predictability.[24] The randomisation algorithm then presented a treatment allocation as either intensive versus guideline BP lowering and, when relevant, intensive versus guideline lipid lowering. Because of the low recruitment rate, the numbers of minimisation variables were reduced during the trial to four (age, ACE-R, systolic BP, total cholesterol). Drugs for patients randomised to intensive treatment were prescribed via the recruiting site’s hospital system, and those randomised to guideline treatment attended their general practitioners for routine treatment.

### Procedures

Treatment was started immediately after randomisation, was given daily for at least 6 months, and consisted of antihypertensive therapy, and lipid lowering therapy in patients with ischaemic stroke. Drugs were sourced locally from any licensed manufacturer and given open-label. Hospital research clinics managed intensive medications with the aim of lowering systolic BP to <125 mmHg and/or LDL-cholesterol <2.0 mmol/l. General practitioners were requested to manage patients randomised to guideline treatment(s) according to national or local guidelines, with the aim of lowering systolic BP <140 mmHg and/or LDL-cholesterol <3.0 mmol/l.[24]

Study drugs were stopped when the patient withdrew consent, for safety reasons, or when unacceptable adverse events developed. All patients received standard care, including oral antithrombotic drugs (in patients with ischaemic stroke) for secondary prevention.

The PODCAST website was used to record demographic and clinical characteristics. Blood pressure was measured twice using a validated automated monitor (OMRON Healthcare Company, Kyoto, Japan) supplied to each site. Sites were asked to provide a trained member of their research staff who was unaware of treatment assignment to do the post-randomisation assessments in hospital clinics. Independent expert assessors, who were masked to treatment assignment, provided central adjudication: cognitive outcomes (AB, CB, GF); vascular events (PP, AM, RH); CT and MRI scans (JW, DB); and SAEs, including cause-specific deaths (NS, TE). Patients who did not receive their assigned treatment or who did not adhere to the protocol were followed in full. The coordinating centre (masked to treatment allocation) did the final central telephone follow-up by October 2014.

### Outcomes

Outcomes were measured both in clinic and via telephone. The primary outcome measure was ACE-R [28, 29] which was assessed at each follow-up clinic. Post stroke cognitive impairment more commonly affects executive dysfunction and for this reason the ACE-R, which incorporates executive and attentional tasks, was chosen. As pre-specified secondary cognition measures, further tests were also administered: Stroop test; Trail-Making Tests A and B; category fluency (animal naming); Mini-Mental State Examination; Telephone Interview for Cognition Scale-Modified (TICS-M [30]); premorbid cognitive function assessed in an informant interview using the IQCODE;[26] and dementia (DSM IV). The ACE-R is a brief cognitive screening tool that is predominantly used to screen for neurodegenerative dementia in memory clinics. The ACE-R has been used in stable cerebrovascular disease (1 year post-stroke/TIA), with optimal sensitivities and specificities for detecting mild cognitive impairment (MCI) achieved at cut-offs between 92 and 94 (ACE-R 92: sensitivity 72%, specificity 79%; ACE-R 94: sensitivity 83%, specificity 73%).[31] The secondary cognition outcomes were chosen to address three competing interests: first, to cover multiple domains (e.g. memory, attentional); second, to be deliverable by telephone; and third, to be in common research use to allow calibration with earlier studies.

Pre-specified non-cognitive secondary outcomes comprised BP; fasting lipids; dependency (mRS);[32] activities of daily living (Barthel index, BI); mood (short Zung depression score [33]); health-related quality of life (European quality of life-5 dimensions, EQ-5D,[34] from which health utility status was calculated [EQ-5D/HUS], and EQ-Visual Analogue Scale); vascular events (stroke, myocardial infarction), health resource utilisation; and disposition. Telephone follow-up included telephone-administered MMSE, TICS-M, mRS, BI, Zung, EQ-5D and EQ-VAS. The safety outcomes were all-cause case fatality, cause-specific case fatality, and serious adverse events.

Clinic measurements were performed and recorded by research nurses/coordinators at each site who were trained (e.g. using videos, start-up meetings). Telephone follow-up was performed centrally, again by trained staff.

### Statistical analyses

The original protocol specified recruitment of 600 patients to test feasibility, tolerability and safety in a start-up phase of a planned larger phase III trial;[24] as such, no formal sample size calculation was performed. However, limited recruitment of participating sites and NHS funding of intensive BP/lipid drugs (e.g. atorvastatin) meant that a new target recruitment of 100 participants was set. Ultimately just 83 patients were enrolled. No formal sample size calculation was performed for the start-up phase.

Treatment intensity was expected to occur through increasing the number of different drugs and their dose (especially to achieve intensive BP lowering), changing tablets to a more powerful drug (especially with statins for intensive lipid lowering), and increasing the dose of tablets up to their maximum. Compliance with these approaches is described both as the number of tablets participants were taking, and the sum of the dose-adjusted number of tablets (sum of tablet dose / maximum licensed dose). By example, the maximum dose of amlodipine is 10 mg so a dose-adjusted value for 5mg is 0.5. Because outcomes such as mRS, EQ-5D-HUS, and Barthel index include death as part of their scale (scores of 6 on mRS, 0 on EQ-5D-HUS, and –5 on the Barthel index), and in case treatment was associated with asymmetric effects on death and other outcome measures (e.g. more death and less impairment), an extreme value for death was added to the other outcome scales with scores: –1 for ACE-R, EQ-VAS, MMSE, MoCA, Stroop accuracy, Stroop correct answers, TICS, Trail making A & B correct answers, animal naming; 301 for Stroop times; 403 for Trail making A time; 601 for Trail making B time and 102·5 on the Zung depression scale, as used previously.[24, 35, 36]

Effects are reported as a mean difference (MD) with 95% CI. We calculated MD and significance with multiple regression on the mean on-treatment score, with adjustment for baseline value, age, systolic BP, total cholesterol, time since stroke and treatment assignment for the other factorial intervention. We assessed the heterogeneity of the treatment effects on the primary outcome in pre-specified subgroups by adding an interaction term to an adjusted multiple regression model. Other analyses used ordinal or binary logistic regression, or Cox proportional hazards regression. The nominal level of significance for all analyses was p<0·05 with no interim analysis. In general, no adjustments were made for multiplicity of testing. We did analyses with SAS version 9.3 according to the intention-to-treat principle.

### Role of the funding source

The trial was funded equally by grants from Alzheimer’s Society and Stroke Association in the UK. There was no commercial support for the trial, and antihypertensive and lipid lowering drugs were prescribed by the responsible physician and sourced locally. The grant applicants conceived and designed the trial and wrote the protocol. Study data were collected, monitored, and analysed by the PODCAST Coordinating Centre in Nottingham, UK. Analysis, interpretation, and report writing were done independently of the funders and sponsor. The corresponding author and two other authors (PS, LW) had full access to all the data in the study; additionally, the corresponding author had final responsibility for the decision to submit for publication, and is the guarantor for the study. This study is registered as ISRCTN85562386 (http://www.isrctn.com/ISRCTN85562386).

## Results

### Trial conduct

**Fig 1:**
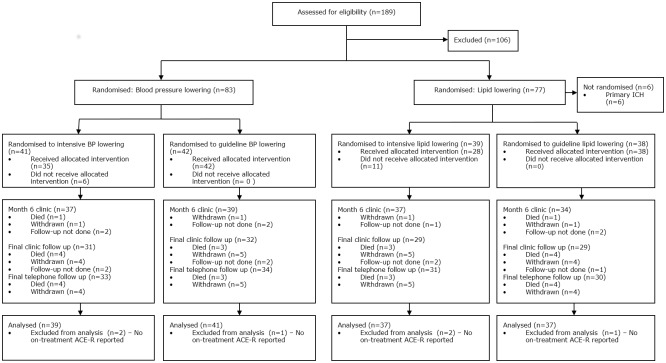
CONSORT flow diagram of patient randomisation, follow up, outcome, and withdrawals. Screening for eligibility was not collected routinely. Data are number/Number (%).

Between 7 October 2010 and 31 January 2014, 83 patients were enrolled from 19 sites in the UK (Fig 1). 41 patients were assigned to the intensive BP lowering group and 42 patients were assigned to the guideline BP group (Table 1). 77 (93%) patients presented with an ischaemic stroke and were included in the intensive lipid lowering (n = 39) or guideline lipid lowering (n = 38) groups of the trial. There were 106 screen failures, the commonest reasons being age between 60–70 and t-MMSE >20 (41%) and systolic BP <125 mmHg (18%).[25] The mean age was 74.0 years, 23% were female, median time from stroke onset to randomisation was 4.5 months, and mean ACE-R score 86.1. The treatment groups were well balanced at baseline (Table 1). Protocol violations at baseline were apparent in 4 participants who were recruited with a mRS >2 (S1 Table). The mean (standard deviation) length of follow-up was 23.9 (8.0) months in those randomised to intensive BP lowering and 24.3 (10.3) months taking guideline medications. 5 participants did not have at least one follow-up performed according to the protocol. During follow-up, 9 patients withdrew from the trial (S2 Table).

**Table 1:** Clinical characteristics at randomisation, by intensive vs guideline blood pressure and lipid lowering.

	All	Intensive BP	Guideline BP	Intensive lipids	Guideline lipids
Number	83	41	42	39	38
Age (years) †	74.0 (6.8)	73.0 (6.5)	75.1 (6.9)	74.2 (6.4)	74.4 (6.9)
Sex, male (%) †	64 (77.1)	33 (80.5)	31 (73.8)	30 (76.9)	31 (81.6)
Time to randomisation [months] †	4.5 [1.3]	4.6 [1.5]	4.4 [1.0]	4.4 [1.4]	4.6 [1.3]
Medical history (%)					
Memory problem	34 (44.2)	16 (41.0)	18 (47.4)	10 (27.8)	20 (57.1)
Hypertension, treated	69 (83.1)	36 (87.8)	33 (78.6)	32 (82.1)	31 (81.6)
Hyperlipidaemia	73 (88.0)	34 (82.9)	39 (92.9)	36 (92.3)	32 (84.2)
Diabetes mellitus	17 (20.5)	12 (29.3)	5 (11.9)	8 (20.5)	8 (21.1)
Atrial fibrillation	15 (18.1)	8 (19.5)	7 (16.7)	10 (25.6)	5 (13.2)
Stroke	8 (9.6)	3 (7.3)	5 (11.9)	3 (7.7)	5 (13.2)
IHD	20 (24.1)	11 (26.8)	9 (21.4)	8 (20.5)	11 (28.9)
PAD	5 (6.0)	1 (2.4)	4 (9.5)	4 (10.3)	1 (2.6)
Smoking, ever (%)	56 (67.5)	31 (75.6)	25 (59.5)	24 (61.5)	29 (76.3)
Alcohol [upw]	3 [0,10]	3 [0,10]	3 [0,8]	5 [1,14]	2 [0,7]
Index stroke ‡					
Ischaemic (%)	77 (92.8)	38 (92.7)	39 (92.9)	39 (100)	38 (100)
Haemorrhagic (%)	6 (7.2)	3 (7.3)	3 (7.1)	0 (0)	0 (0)
Side, right weakness (%)	30 (47.6)	15 (45.5)	15 (50.0)	19 (59.4)	10 (35.7)
Dysphasia (%) †	23 (27.7)	12 (29.3)	11 (26.2)	9 (23.1)	11 (28.9)
BP drugs, number †					
Mode	2	1	2	2	1
Median	2 [1,2]	2 [1,2]	2 [1,2]	2 [1,2]	2 [1,2]
Mean	1.8 (0.9)	1.7 (1.0)	1.9 (0.8)	1.8 (0.9)	1.8 (0.9)
>0 (%)	69 (92.0)	35 (85.4)	34 (100)	31 (93.9)	32 (88.9)
BP drug classes (%)					
ACE-I or ARB	39 (52.0)	20 (48.8)	19 (55.9)	19 (57.6)	18 (50.0)
ß-receptor antagonist	18 (24.0)	8 (19.5)	10 (29.4)	10 (30.3)	6 (16.7)
Calcium channel blocker	39 (52.0)	21 (51.2)	18 (52.9)	16 (48.5)	20 (55.6)
Diuretic	20 (26.7)	7 (17.1)	13 (38.2)	10 (30.3)	7 (19.4)
Other	6 (8.0)	2 (4.9)	4 (11.8)	4 (12.1)	1 (2.8)
Lipid tablets (%)					
Any statin †	79 (95.2)	40 (97.6)	39 (92.9)	38 (97.4)	37 (97.4)
Fluvastatin	1 (1.3)	0 (0)	1 (2.6)	1 (2.6)	0 (0)
Simvastatin	55 (69.6)	25 (62.5)	30 (76.9)	27 (71.1)	26 (70.3)
Atorvastatin	21 (26.6)	13 (32.5)	8 (20.5)	10 (26.3)	9 (24.3)
Rosuvastatin	2 (2.5)	2 (5.0)	0 (0)	0 (0)	2 (5.4)
Ezetimibe	1 (1.2)	0 (0)	1 (2.4)	1 (2.6)	0 (0)
Pre-morbid mRS <2 (%) †	60 (72.3)	26 (63.4)	34 (81.0)	27 (69.2)	28 (73.7)
ACE-R (/100) †	86.1 (7.7)	85.7 (8.1)	86.5 (7.4)	87.6 (7.7)	84.5 (7.3)
NIHSS (/42)	0.77 (1.1)	0.80 (1.1)	0.74 (1.1)	0.59 (0.9)	0.97 (1.3)
TACS (%)	6 (7.2)	3 (7.3)	3 (7.1)	4 (10.3)	2 (5.3)
Systolic BP (mmHg) †	147.1 (18.6)	145.9 (19.8)	148.3 (17.5)	147.6 (20.6)	147.9 (16.7)
<140 (%)	32 (38.6)	16 (39.0)	16 (38.1)	16 (41.0)	14 (36.8)
<125 (%)	9 (10.8)	6 (14.6)	3 (7.1)	5 (12.8)	3 (7.9)
Diastolic BP (mmHg)	82.1 (11.1)	82.5 (11.7)	81.7 (10.7)	83.3 (11.9)	81.2 (10.2)
Heart rate (bpm)	71.5 (14.2)	71.7 (14.9)	71.3 (13.8)	72.9 (16.4)	70.4 (11.5)
Lipids (mmol/l)					
Total cholesterol †	4.0 (0.8)	3.9 (0.7)	4.1 (1.0)	4.0 (0.6)	4.0 (1.0)
Triglycerides	1.3 (0.6)	1.3 (0.6)	1.3 (0.6)	1.4 (0.7)	1.3 (0.6)
HDL-cholesterol	1.4 (0.5)	1.3 (0.4)	1.5 (0.6)	1.4 (0.4)	1.5 (0.6)
LDL-cholesterol	2.0 (0.7)	2.0 (0.5)	2.0 (0.8)	2.0 (0.5)	2.0 (0.8)
Non-HDL-cholesterol	2.6 (0.8)	2.6 (0.7)	2.6 (0.9)	2.6 (0.6)	2.5 (1.0)

Data are number (%), median [interquartile range] or mean (standard deviation).

^†^ Minimisation variable–from June 2013 limited to age, ACE-R, systolic blood pressure and total cholesterol.

^‡^ Stratification variable;

^¶^ Protocol violation

Non-HDL-cholesterol = total cholesterol–HDL-cholesterol. ACE-R: Addenbrooke’s Cognitive Examination-revised; BP: blood pressure; bpm: beats per minute; HDL: high density lipoprotein; IHD: ischaemic heart disease; LDL: low density lipoprotein; mRS: modified Rankin Scale; NIHSS: National Institutes of Health Stroke Scale; PAD: peripheral arterial disease; IHD: current angina or previous angina or myocardial infarction; TACS: Total anterior circulation syndrome.

### Drug management

The number of antihypertensive tablets in patients randomised to intensive BP lowering rose from 1.7 at baseline to 2.0 during follow-up, this including 6 participants who were randomised to intensive BP lowering but did not receive it (S1 Table). However, the dose adjusted number of tablets increased from 1.2 to about 1.7. In patients randomised to guideline BP therapy the number of tablets, and dose-adjusted number of tablets were static at 1.9 and 1.2 respectively. The number of tablets in patients randomised to intensive lipid lowering rose from 1.1 to about 1.4 (including 11 participants who did not receive intensive lipid lowering, S1 Table), with a bigger rise in dose-adjusted number of tablets from 0.8 to about 1.3. In patients randomised to guideline lipid therapy the number of tablets, and dose-adjusted number of tablets, did not change at 1.0 and 0.5 respectively (S3 Table).

### Haemodynamic measures

**Fig 2:**
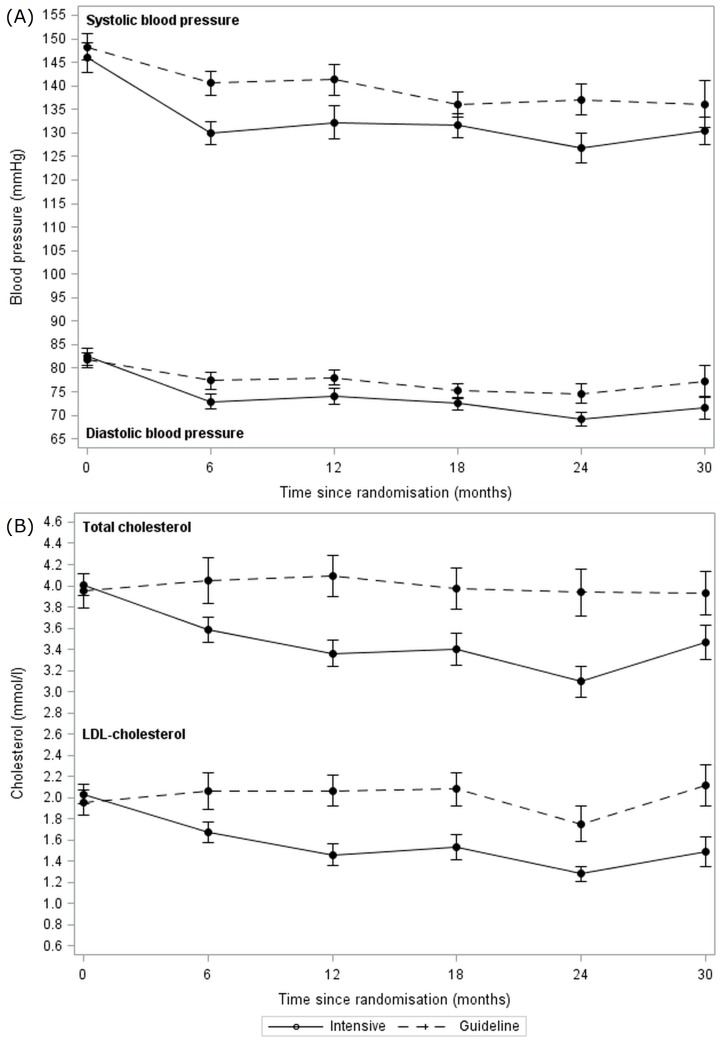
Changes during follow-up in blood pressure and cholesterol by intensive vs guideline groups. **Data are mean and standard error of mean**. (A) Systolic and diastolic blood pressure by intensive vs guideline blood pressure lowering groups. (B) Total cholesterol and LDL-cholesterol by intensive vs guideline lipid lowering groups.

The mean BP at baseline was 147/82 mmHg and fell in both intensive and guideline BP lowering groups during treatment (Fig 2A). Most of the BP reduction occurred during the first 6 months of treatment, by which time BP was lower in those patients randomised to the intensive BP group by 10.6/5·5 mmHg (2p<0·001/0·004) (S4 Table). The difference in systolic BP between treatment groups exceeded the trial’s target of 10 mmHg. The reduction in BP was further confirmed in 18 patients who had ambulatory BP monitoring at selected sites, with BP lower by 10.0/6.3 mmHg in those randomised to intensive BP lowering (S5 Table). The target systolic BP levels of <125 mmHg in the intensive group and <140 mmHg in the control group were achieved by only 17 (45.9%, S6 Table) and 22 (55.0%) participants respectively. Heart rate was 5·0 beats per min lower in the intensive BP group than in the guideline group at 6 months (2p = 0·034, S4 Table).

### Lipid measures

The mean total and LDL-cholesterol at baseline was 4.0/2.0 mmol/l and fell over 12 months on treatment in the intensive but not guideline lipid-lowering group (Fig 2B). Mean total, LDL and non-HDL cholesterol levels differed between the treatment groups at 6 months after randomisation, and were lower by 0.54/0.44/0.44 mmol/l (2p = 0·003/0·003/0.024) in the intensive group (S4 Table); potential explanations are given in S6 Table. The difference in LDL-c between treatment groups was just over half the intended 1.0 mmol/l. The target LDL-cholesterol levels of <1.3 mmol/l in the intensive group and <3.0 mmol/l in the control group were achieved by 10 (28.6%) and 28 (87.5%) participants respectively.

### Clinical and safety outcomes

**Fig 3:**
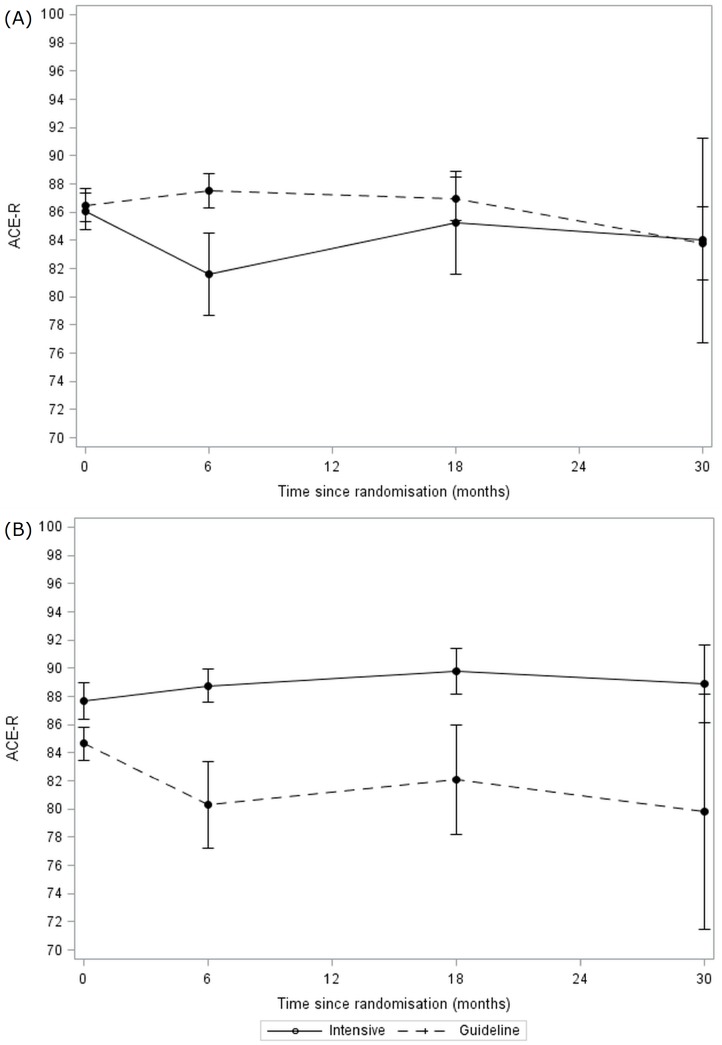
Changes in Addenbrooke’s Cognitive Examination-Revised score during follow-up for intensive vs guideline groups. **Data are mean and standard error of mean**. (A) Intensive vs guideline blood pressure lowering. (B) Intensive vs guideline lipid lowering.

80 (96%) patients had both vital status and the primary outcome recorded on at least one occasion during treatment (Fig 1). Final follow-up was performed between 6 and 48 months, with a median of 24 months (mean 24). When adjusted for randomisation variables, the primary outcome (ACE-R) scores in the intensive BP lowering group and the guideline group did not differ during treatment (MD in intensive BP lowering group lower by 3.6, 95% CI -9.7 to 2.4, p = 0·24) (Table 2, Fig 3A, S7 Table). The effects of the treatments were consistent across all pre-specified subgroups. There were no differences in secondary outcomes including cognition scores (MMSE, MoCA, TICS, trail making, Stroop, IQ-CODE, animal naming), rates of dementia and vascular events, and functional outcome (mRS, BI), mood (ZDS) and quality of life (HUS, EQ-VAS) (Table 2, S1 Fig). The overall rate of serious adverse events, fatal events, and dementia and vascular events occurring by end of trial did not differ between the two BP groups (S8–S10 Tables). The overall rate of dementia (2 cases) appeared lower than expected (S6 & S9 Tables).

**Table 2:** Primary and secondary cognition and other functional measures by treatment group: intensive vs guideline blood pressure lowering. Comparison by multiple regression of mean on-treatment score with adjustment for baseline value and age, systolic blood pressure, total cholesterol, time since stroke, treatment assignment (intensive vs lipid guideline lowering vs none).

	Baseline	Intensive	Guideline	Mean difference (95% CI)	p
Follow-up (months)	-	23.9 (8.0)	24.3 (10.3)		
Clinic					
ACE-R	86.1 (7.7)	80.8 (21.6)	84.4 (12.5)	-3.6 (-9.7, 2.4)	0.24
MMSE	27.9 (2.0)	26.0 (6.5)	27.0 (3.8)	-1.2 (-3.3, 0.8)	0.23
MoCA	24.0 (2.6)	22.6 (6.2)	23.2 (3.6)	-0.7 (-2.6, 1.2)	0.46
TICS	24.1 (4.2)	22.1 (6.8)	23.2 (5.1)	-1.8 (-3.8, 0.3)	0.086
Trail					
A time (seconds)	57.1 (30.0)	79.1 (88.0)	68.5 (46.9)	13.2 (-13.6, 39.9)	0.33
A correct answers	24.6 (2.8)	22.9 (5.4)	24.1 (3.0)	-1.4 (-3.1, 0.4)	0.13
B time (seconds)	155.5 (90.8)	178.4 (131.7)	165.8 (97.4)	6.9 (-27.5, 41.2)	0.70
B correct answers	22.1 (5.5)	21 (6.6)	22.0 (4.6)	-1.6 (-3.8, 0.7)	0.17
Stroop					
1 accuracy	23.3 (1.1)	21.5 (6.2)	22.6 (3.1)	-1.5 (-3.4, 0.4)	0.12
1 time (seconds)	54.7 (20.6)	68.1 (65.2)	59.9 (39.0)	11.1 (-10.1, 32.2)	0.31
2 accuracy	23.4 (1.1)	21.9 (5.8)	22.7 (2.9)	-1.2 (-3.1, 0.7)	0.20
2 time (seconds)	46.1 (20.7)	61.0 (63.8)	51.1 (41.4)	9.7 (-12.7, 32.1)	0.40
3 accuracy	20.0 (5.3)	18.4 (6.4)	19.3 (5.8)	-1.4 (-3.7, 0.9)	0.23
3 time (seconds)	66.9 (34.5)	80.0 (61.9)	72.4 (50.3)	6.6 (-16.5, 29.8)	0.58
Interference accuracy	-3.4 (5.4)	-5.3 (6.6)	-4.2 (5.6)	-1.5 (-3.8, 0.9)	0.22
Interference time (seconds)	20.9 (22.1)	32.8 (44.2)	27.5 (29.9)	6.8 (-8.5, 22.0)	0.38
Informant (IQ-code)	3.0 (0.5)	3.1 (0.5)	3.0 (0.5)	0.1 (-0.1, 0.3)	0.23
Knafelc [37]	11.1 (10.0)	14.5 (20.0)	10.5 (9.7)	2.7 (-2.4, 7.8)	0.30
Animal naming	15.8 (5.5)	15.7 (6.7)	15.6 (5.2)	1.2 (-1.0, 3.4)	0.28
Telephone					
MMSE	20.4 (1.8)	19.1 (5.3)	19.3 (4.9)	-0.4 (-2.5, 1.7)	0.73
TICS	24.1 (4.2)	23.2 (7.4)	23.7 (7.3)	-1.1 (-3.9, 1.7)	0.43
Function					
mRS	1.1 (0.8)	1.3 (1.3)	1.4 (1.0)	0.0 (-0.4, 0.5)	0.90
Barthel Index	97.9 (4.8)	91.1 (22.7)	91.9 (13.4)	-2.1 (-9.6, 5.4)	0.58
HUS (EQ-5D)	0.8 (0.2)	0.7 (0.2)	0.7 (0.2)	0.0 (-0.1, 0.1)	0.91
EQ-VAS	72.9 (17.6)	69.0 (22.0)	73.2 (14.5)	-4.0 (-10.7, 2.7)	0.24
ZDS	45.6 (12.6)	47.5 (14.6)	45.9 (13.0)	3.1 (-1.8, 8.0)	0.22

ACE-R: Addenbrooke’s Cognitive Examination-R; EQ-VAS: EuroQoL-Visual Analogue Scale; HUS: Health Utility Status (from EuroQoL 5-dimensions); MMSE: Mini-Mental State Examination; MoCA: Montreal Cognitive Assessment; mRS: modified Rankin Scale; TICS: Telephone Interview Cognition Scale; t-MMSE: telephone-Mini-Mental State Examination; ZDS: Zung Depression Scale.

Patients who died were assigned the following scores: -5: BI; -1: ACE-R, EQ-VAS, MMSE, MoCA, Stroop accuracy, TICS, Trail making A & B correct answers, animal naming; 0: EQ-5D/HUS; 6: mRS; 102.5: ZDS; 301 secs: Stroop time alive censored at 300 secs; 403: Trail making A time; 601: Trail making B time.

There was no difference in the primary outcome (ACE-R) during treatment between patients randomised to intensive or guideline lipid lowering (MD in intensive lipid lowering group higher by 4.4, 95% CI -2.1 to 10.9, p = 0·18) (Table 3, Fig 3B). However, in a *post hoc* analysis, when comparing ACE-R scores at specific clinic visits, ACE-R was significantly higher by 8.4 points (p = 0.032) at 6 months in patients randomised to intensive lipid lowering (Fig 3B, S7 Table). The effects of the treatments were consistent across all pre-specified subgroups. The intensive lipid group had significantly higher cognition scores judged using Trail Making, category fluency (animal naming) and Stroop accuracy (Table 3). Additionally, the mRS was significantly lower, and EQ-VAS higher (S2 Fig), in patients randomised to intensive lipid lowering. Although the total number of serious adverse events did not differ between the groups, chest infections or pneumonia were less common in patients taking intensive treatment (S8 Table). Rates of death, and dementia and vascular events did not differ between the groups (S9 & S10 Tables).

**Table 3:** Primary and secondary cognition and other functional measures by treatment group: intensive vs guideline lipid lowering. Comparison by multiple regression of mean on-treatment score with adjustment for baseline value, age, systolic blood pressure, total cholesterol, time since stroke and treatment assignment (intensive vs guideline BP lowering).

Outcome	Baseline	Intensive	Guideline	Mean difference (95% CI)	p
Follow-up (months)	-	24.6 (10.6)	22.9 (7.8)		
Clinic					
ACE-R	86.0 (7.6)	86.5 (11.7)	78.2 (22.2)	4.4 (-2.1, 10.9)	0.18
MMSE	27.9 (1.9)	27.6 (3.2)	25.3 (6.9)	1.9 (-0.3, 4.1)	0.085
MoCA	24.1 (2.6)	24.1 (3.4)	21.8 (6.3)	1.2 (-0.9, 3.2)	0.26
TICS	24.3 (4.2)	23.9 (4.5)	21.4 (7.4)	1.3 (-0.9, 3.5)	0.25
**Trail A (time), (seconds)**	**58.1 (30.7)**	**60.2 (41.1)**	**89.6 (91.4)**	**-28.9 (-56.7, -1.1)**	**0.045**
Trail A (correct answers)	24.6 (2.9)	24.0 (2.9)	22.8 (5.7)	1.1 (-0.8, 2.9)	0.25
Trail B (time), (seconds)	151.5 (78.9)	149.3 (81.6)	195.7 (141.8)	-25.8 (-61.2, 9.6)	0.15
Trail B (correct answers)	**21.9 (5.7)**	**22.8 (3.7)**	**20.3 (7.0)**	**2.7 (0.3, 5.0)**	**0.025**
Informant (IQ-code)	3.0 (0.5)	3.0 (0.5)	3.1 (0.5)	-0.1 (-0.3, 0.1)	0.43
Knafelc	10.9 (9.9)	9.0 (7.7)	16.9 (21.2)	-5.0 (-10.3, 0.3)	0.067
**Animal naming**	**15.8 (5.4)**	**17.5 (5.4)**	**14.0 (6.1)**	**3.0 (0.8, 5.3)**	**0.008**
Stroop					
1 accuracy	23.2 (1.2)	22.9 (2.7)	20.9 (6.7)	1.7 (-0.3, 3.8)	0.092
1 time (seconds)	54.6 (20.5)	53.3 (29.5)	77.0 (71.9)	-17.3 (-39.8, 5.3)	0.13
2 accuracy	23.4 (1.1)	23.0 (2.5)	21.4 (6.3)	1.4 (-0.6, 3.4)	0.16
2 time (seconds)	46.0 (19.9)	47.9 (28.0)	66.1 (73.5)	-15.6 (-38.9, 7.6)	0.19
**3 accuracy**	**20.0 (5.2)**	**20.5 (4.1)**	**17.0 (7.5)**	**2.7 (0.3, 5.1)**	**0.027**
3 time (seconds)	67.3 (34.8)	66.0 (31.9)	87.6 (74.3)	-20.5 (-44.2, 3.1)	0.088
**Interference accuracy**	**-3.4 (5.3)**	**-3.1 (4.1)**	**-6.5 (7.6)**	**2.7 (0.2, 5.1)**	**0.033**
Interference time (seconds)	21.3 (22.3)	23.4 (23.7)	38.2 (49.1)	-14.0 (-29.8, 1.8)	0.082
Telephone					
MMSE	20.4 (1.7)	20.1 (4.0)	18.2 (6.1)	1.7 (-0.4, 3.9)	0.12
TICS	24.3 (4.2)	25.0 (6.4)	21.7 (8.2)	2.1 (-0.9, 5.1)	0.17
Function					
**mRS**	**1.1 (0.8)**	**1.1 (0.9)**	**1.6 (1.4)**	**-0.5 (-1.0, 0.0)**	**0.036**
Barthel Index	98.1 (4.1)	95.1 (12.0)	87.2 (23.6)	7.5 (-0.5, 15.4)	0.067
HUS (EQ-5D)	0.8 (0.2)	0.8 (0.2)	0.7 (0.2)	0.1 (0.0, 0.2)	0.084
**EQ-VAS**	**73.0 (17.8)**	**75.3 (14.9)**	**65.9 (21.4)**	**8.6 (1.7, 15.5)**	**0.014**
ZDS	45.6 (12.7)	45.5 (12.0)	48.4 (16.2)	-3.0 (-8.2, 2.1)	0.25

ACE-R: Addenbrooke’s Cognitive Examination-R; EQ-VAS: EuroQoL-Visual Analogue Scale; HUS: Health Utility Status (from EuroQoL 5-dimensions); MMSE: Mini-Mental State Examination; MoCA: Montreal Cognitive Assessment; mRS: modified Rankin Scale; TICS: Telephone Interview Cognition Scale; t-MMSE: telephone-Mini-Mental State Examination; ZDS: Zung Depression Scale.

Patients who died were assigned the following scores: -5: BI; -1: ACE-R, EQ-VAS, MMSE, MoCA, Stroop accuracy, TICS, Trail making A & B correct answers, animal naming; 0: EQ-5D/HUS; 6: mRS; 102.5: ZDS; 301 secs: Stroop time alive censored at 300 secs; 403: Trail making A time; 601 Trail making B time

In a qualitative end-of-trial assessment of patient views about the study, a number of themes were expressed including having enjoyed being in the trial, hope that the results would help others, sadness that the trial did not continue (as originally planned), and thanks for being in the trial (S11 Table).

## Discussion

This small partial factorial internal pilot trial in patients with recent stroke found that it was feasible to lower BP intensively, and that intensive antihypertensive treatment, in comparison with guideline BP lowering, reduced BP by around 11/6 mmHg but did not alter cognitive impairment, as assessed using the ACE-R. Additionally there were no differences in other measures of cognition, functional outcome, or vascular events. Intensive lipid lowering was partially effective in comparison with guideline lowering, and reduced total and LDL-cholesterol by around 0.5/0.4 mmol/l but did not alter ACE-R. Nevertheless, intensive lipid therapy was associated with significant improvements in some secondary measures, including cognition (ACE-R at 6 months of treatment, trail making, category fluency, Stroop accuracy), function (mRS), and quality of life (EQ-VAS).

The potential mechanisms by which intensive BP lowering might benefit cognition are multiple, including reducing the risk of recurrent stroke, and increasing cerebral blood flow and so reducing the risk of hypotensive/low perfusion cerebral damage.[38] Lowering BP has been associated with less cognitive impairment in several trials involving differing patient groups,[4] including those with mid-life hypertension or chronic stroke. However, the results have been inconsistent and assumed a linear relationship between change in BP and cognition. Since the relationship between BP and stroke recurrence may be curvilinear or ‘J-shaped’,[39] the same may also be true for the relationship between BP and cognition.[4] If so, a trial testing intensity of BP lowering might achieve on-treatment BP levels that straddle the optimum BP level, or nadir of the relationship between BP and cognition; in this case, differences in cognition would not be expected between more and less intensive BP lowering. The neutral PODCAST results are compatible with this hypothesis with the nadir lying in the systolic BP range of 130–140 mmHg although the exact level presumably varies by patient and stroke type.

Evidence that lowering cholesterol levels reduces cognitive impairment and dementia is missing since both large and small trials have not identified any benefit.[4, 19, 20] PODCAST confirms that intensive lipid lowering did not alter the primary measure of cognition. Nevertheless, a number of secondary outcomes were positive in favour of intensive lipid lowering (following adjustment for baseline scores). Vascular cognitive impairment more commonly affects executive function and sub-cortical processes such as response times rather than episodic memory and praxis (as seen in Alzheimer’s disease); in this context, intensive lipid lowering was associated with better scores on tests assessing executive function and attention, including faster trail making (test A) and better accuracy on the Stroop, tests that identify cognitive impairment in post stroke populations[40]

If lipid lowering does limit cognitive impairment and clinical dysfunction post stroke then it is likely that the effect is mediated through statin intensity since differences in type and dose of statin were the main difference between the treatment groups. In addition to lowering total-c, LDL-c and non-HDL-c levels, statins have multimodal effects mediated through enhancing endothelial nitric oxide synthase activity (with benefit on vascular integrity and function), and attenuating inflammation, and smooth muscle cell and platelet function.[41, 42] In addition to reducing vascular events, including stroke recurrence, other potential clinical effects of statins include reducing dementia, cognitive impairment and pneumonia.[23]

PODCAST has several important limitations. First, it was far smaller than intended (83 of a planned 600 participants). The low sample size was largely due to problems in recruiting sufficient primary care and hospital sites due to a reluctance by healthcare commissioners to pay for intensive lipid lowering drugs such as atorvastatin (this drug was on patent when the trial commenced). This and other impediments to recruitment are discussed in a previous publication.[25] The funding of excess treatment costs (treatment-related costs in a clinical trial that would continue after the trial finished if the treatment was effective) remains a significant issue in the UK and this can delay the start of trials, and delay and limit recruitment.[43] In this respect, the protocol could not be delivered but removal of this impediment related to excess treatment costs might allow a similar protocol to be tested in a different funding environment. Second, the small sample size meant that the study was very underpowered to detect worthwhile clinical benefits. The neutral findings for the effect of intensive BP and lipid lowering could simply reflect a false neutral finding (type II error) such that intensive lowering might actually be beneficial. Although some secondary analyses were positive in favour of intensive lipid lowering, these might reflect a false positive finding (type I error). Since secondary analyses were necessarily hypothesis generating, we did not adjust for multiplicity of testing. Analyses were adjusted statistically for baseline stratification and minimisation factors so minor differences in cognition scores at baseline are unlikely to have explained the positive secondary findings.

Third, although the target difference in mean systolic BP between the intensive and guideline groups of 10 mmHg was achieved at 6 months, this was not maintained long-term and the intensive group did not reach the absolute target of mean systolic BP <125 mmHg (S6 Table). Treatment was not escalated by hospital investigators in the intensive group, manifest as a failure to increase the number of tablets and dose-adjusted number of tablets. A common concern among investigators was whether the risk of falls would increase with intensive BP lowering although this was not apparent in SAE rates in PODCAST and has not been observed in other trials of intensive BP lowering. Fourth, neither of the two lipid targets were met; the difference in LDL-cholesterol between the two groups was 50–60% of planned. Originally, the target for the intensive group was LDL-cholesterol <2.0 mmol/l but this was reduced to <1.3 mmol/l when it became clear that the average LDL-c at baseline was already achieving target. Nevertheless, the new target was not achieved. Last, although some patients were followed for more than two years, others were not and hence treatment may not have been given for long enough.

In summary, intensive BP and lipid lowering appeared to have acceptable safety in those patients where this was achieved but did not alter cognition in a population with a mean age of 74 with recent stroke and normal/near-normal cognition. The association between intensive lipid lowering and improved scores for some secondary outcomes suggests further trials of intensive lipid lowering are warranted.

## Supporting Information

S1 File CONSORT checklist. (DOCX)

S2 File PODCAST trial protocol, version 16. (PDF)

S1 Fig Euro-Quality of Life-Visual Analogue Scale score during follow-up, by treatment group: intensive vs guideline blood pressure lowering. Data are mean and standard error of mean.
(TIF)

S2 Fig Euro-Quality of Life-Visual Analogue Scale score during follow-up, by treatment group: intensive vs guideline lipid lowering. Data are mean and standard error of mean.
(TIF)

S1 Table Protocol violations, by treatment group. **Data are number (%)**. † A patient whose number of tablets and proportion of maximum dose are not more than their baseline at any follow up.
(DOCX)

S2 Table Withdrawals and reasons for 9 patients. (DOCX)

S3 Table Adherence to BP and lipid lowering therapy through the trial, by intensive versus guideline blood pressure lowering, and by intensive versus guideline lipid lowering. **Data are number of tablets, and number adjusted for dose as proportion of maximum dose**. Dose adjusted: Sum of tablet dose / maximum licensed dose (e.g. amlodipine 5mg is 0.5).
(DOCX)

S4 Table Blood pressure and lipids levels at baseline and months 1, 2, 3 and 6, by treatment group: intensive (BP: n = 37, Lipid: n = 35) vs guideline (BP: n = 40, Lipid: n = 32). **Data are mean (standard deviation) at 0–6 months; comparison by ANCOVA with mean difference adjusted for baseline**. DBP: diastolic blood pressure; G: guideline; HDL: high density; HR: heart rate; I: intensive; LDL: low density; SBP: systolic blood pressure; TC: total cholesterol; TG: triglycerides.
(DOCX)

S5 Table 24 hours ambulatory systolic and diastolic blood pressure and heart rate at baseline and on treatment (usually at 6 months), by treatment group: intensive (n = 6) vs guideline (n = 12). Data are mean (standard deviation); comparison by ANCOVA with mean difference on treatment adjusted for baseline.
(DOCX)

S6 Table Issues during treatment with the trial. (DOCX)

S7 Table Addenbrooke’s Cognitive Examination-Revised at baseline and months 6, 18 and 30, by treatment group: intensive versus guideline blood pressure lowering, and intensive versus guideline lipid lowering. Data are mean (standard deviation). Comparisons by t-test with Bonferroni adjustment for multiple comparisons.
(DOCX)

S8 Table Number of patients with one or more serious adverse events during treatment by organ class (plus selected events within organ classes) by treatment group: intensive vs guideline blood pressure lowering, and intensive lipid vs guideline lowering. **Data are number (%). Comparison by Chi-square test**. † p<0.05.
(DOCX)

S9 Table Fatal events during treatment, by treatment group. Data are number.
(DOCX)

S10 Table Dementia and vascular events during treatment, by treatment group. Data are number of events.
(DOCX)

S11 Table Closing comments from 22 trial participants. (DOCX)
