# Supplementary figures and images for: Intensive versus Guideline Blood Pressure and Lipid Lowering in Patients with Previous Stroke: Main Results from the Pilot ‘Prevention of Decline in Cognition after Stroke Trial’ (PODCAST) Randomised Controlled Trial
Source: PLoS One. 2017 Jan 17;12(1):e0164608. doi: 10.1371/journal.pone.0164608 (PMC5240987; doi:10.1371/journal.pone.0164608)

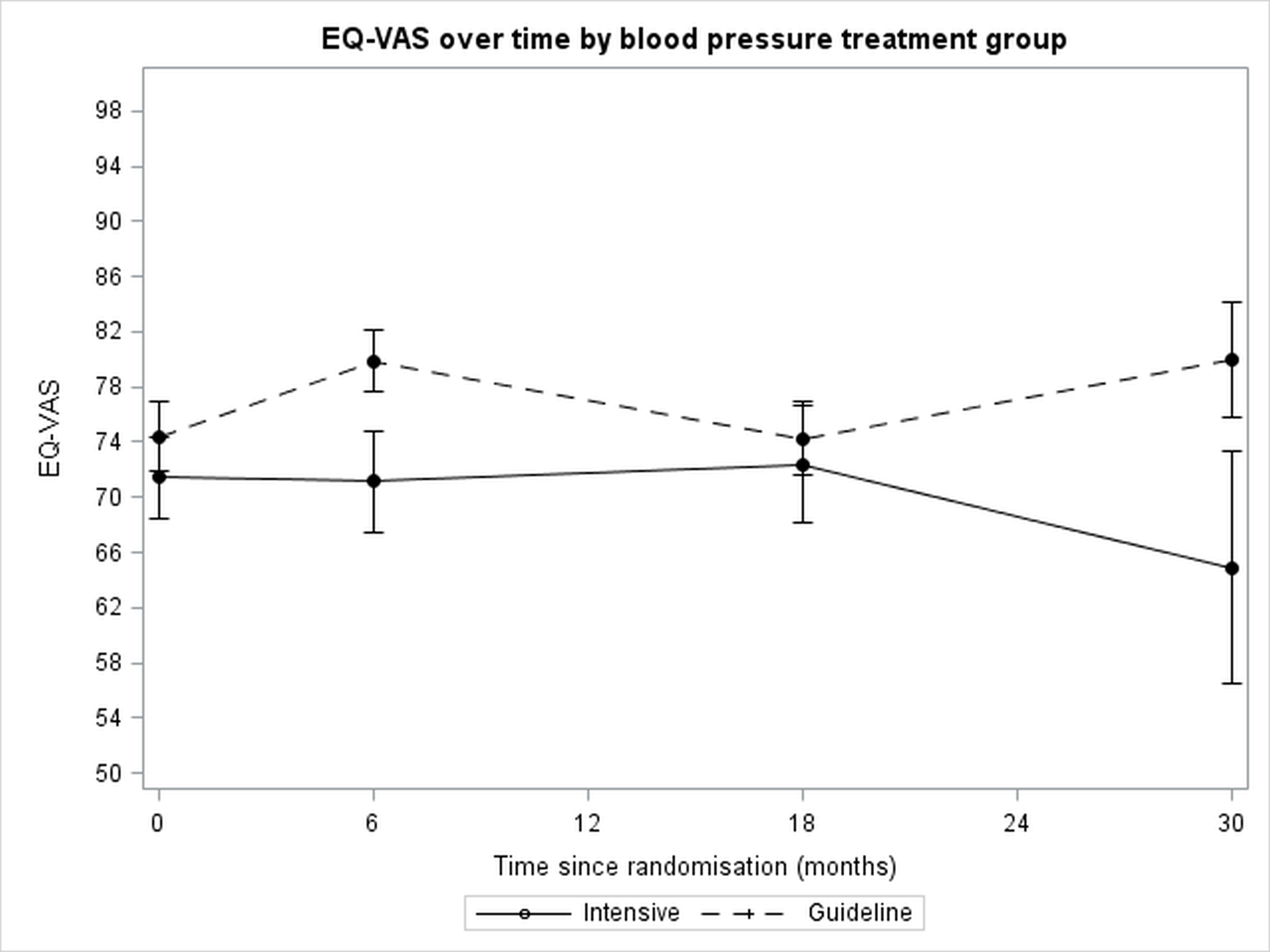

Supplement: S1 Fig — Data are mean and standard error of mean. (TIF) [file pone.0164608.s003.tif]

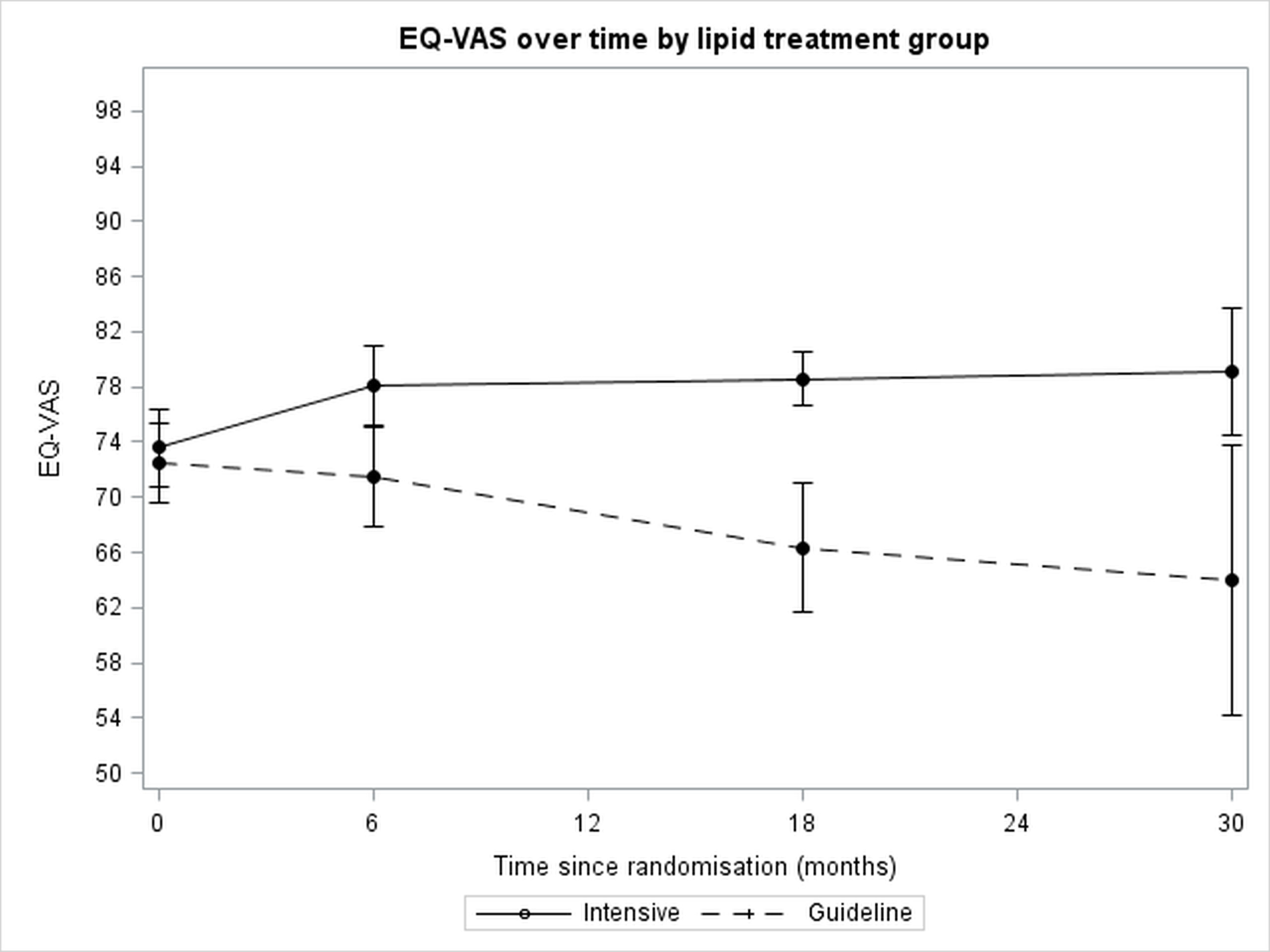

Supplement: S2 Fig — Data are mean and standard error of mean. (TIF) [file pone.0164608.s004.tif]
