# Supplementary material for: Intensive versus Guideline Blood Pressure and Lipid Lowering in Patients with Previous Stroke: Main Results from the Pilot ‘Prevention of Decline in Cognition after Stroke Trial’ (PODCAST) Randomised Controlled Trial
Source: PLoS One. 2017 Jan 17;12(1):e0164608. doi: 10.1371/journal.pone.0164608 (PMC5240987; doi:10.1371/journal.pone.0164608)
Supplement: S1 Table — Data are number (%). † A patient whose number of tablets and proportion of maximum dose are not more than their baseline at any follow up. (DOCX) [file pone.0164608.s005.docx]

|  | All | BP | lowering | Lipid | lowering |
| --- | --- | --- | --- | --- | --- |
|  |  | Intensive | Guideline | Intensive | Guideline |
| Inclusion criteria |  |  |  |  |  |
| mRS >2 | 4 (4.8) | 2 (4.9) | 2 (4.8) | 0 | 4 (10.5) |
| On treatment |  |  |  |  |  |
| Follow-up not performed | 5 (6.0) | 3 (7.3) | 2 (4.8) | 3 (7.7) | 2 (5.3) |
| Treatment |  |  |  |  |  |
| Not given intensive BP lowering † | 6 (14.6) | 6 (14.6) | - | - | - |
| Not given intensive lipid lowering | 11 (28.2) | - | - | 11 (28.2) | - |
| Total number of patients | 25 (30.1) | 11 (26.8) | 4 (9.5) | 13 (33.3) | 6 (15.8) |
