# Supplementary material for: Intensive versus Guideline Blood Pressure and Lipid Lowering in Patients with Previous Stroke: Main Results from the Pilot ‘Prevention of Decline in Cognition after Stroke Trial’ (PODCAST) Randomised Controlled Trial
Source: PLoS One. 2017 Jan 17;12(1):e0164608. doi: 10.1371/journal.pone.0164608 (PMC5240987; doi:10.1371/journal.pone.0164608)
Supplement: S2 Table — (DOCX) [file pone.0164608.s006.docx]

| Reason | Number |
| --- | --- |
| Visit frequency too high | 3 |
| Fatigue | 1 |
| Memory loss/dementia | 1 |
| Terminal condition | 1 |
| Cognitive assessments induced anxiety | 1 |
| Patient moved out of area | 1 |
| Lost to follow-up | 1 |
| Total | 9 |
