# Supplementary material for: Intensive versus Guideline Blood Pressure and Lipid Lowering in Patients with Previous Stroke: Main Results from the Pilot ‘Prevention of Decline in Cognition after Stroke Trial’ (PODCAST) Randomised Controlled Trial
Source: PLoS One. 2017 Jan 17;12(1):e0164608. doi: 10.1371/journal.pone.0164608 (PMC5240987; doi:10.1371/journal.pone.0164608)
Supplement: S3 Table — Data are number of tablets, and number adjusted for dose as proportion of maximum dose. Dose adjusted: Sum of tablet dose / maximum licensed dose (e.g. amlodipine 5mg is 0.5). (DOCX) [file pone.0164608.s007.docx]

| Month |  |  | 0 | 1 | 2 | 3 | 6 | 12 | 18 | 24 | 30 | 36 |
| --- | --- | --- | --- | --- | --- | --- | --- | --- | --- | --- | --- | --- |
| BP | Patients |  | 83 | 40 | 39 | 39 | 76 | 68 | 65 | 44 | 23 | 11 |
|  | Tablets | Intensive | 1.7 | 2.0 | 2.1 | 2.3 | 2.0 | 1.9 | 2.0 | 2.0 | 2.5 | 2.0 |
|  |  | Guideline | 1.9 | - | - | - | 2.0 | 2.0 | 2.0 | 2.2 | 2.0 | 2.3 |
|  | Dose-adjusted | Intensive | 1.2 | 1.3 | 1.5 | 1.5 | 1.4 | 1.7 | 1.7 | 1.9 | 1.6 | 1.3 |
|  |  | Guideline | 1.2 | - | - | - | 1.2 | 1.2 | 1.2 | 1.3 | 1.0 | 1.0 |
| Lipids |  | Patients | 77 | - | - | 38 | 71 | 62 | 59 | 39 | 21 | 9 |
|  | Tablets | Intensive | 1.1 | - | - | 1.3 | 1.3 | 1.5 | 1.3 | 1.3 | 1.3 | 1.7 |
|  |  | Guideline | 1.0 | - | - | - | 1.0 | 1.0 | 1.0 | 1.0 | 1.0 | 1.0 |
|  | Dose-adjusted | Intensive | 0.8 | - | - | 1.0 | 1.2 | 1.3 | 1.4 | 1.2 | 1.1 | 1.3 |
|  |  | Guideline | 0.5 | - | - | - | 0.4 | 0.4 | 0.4 | 0.4 | 0.5 | 0.4 |
