# Supplementary material for: Intensive versus Guideline Blood Pressure and Lipid Lowering in Patients with Previous Stroke: Main Results from the Pilot ‘Prevention of Decline in Cognition after Stroke Trial’ (PODCAST) Randomised Controlled Trial
Source: PLoS One. 2017 Jan 17;12(1):e0164608. doi: 10.1371/journal.pone.0164608 (PMC5240987; doi:10.1371/journal.pone.0164608)
Supplement: S4 Table — Data are mean (standard deviation) at 0–6 months; comparison by ANCOVA with mean difference adjusted for baseline. DBP: diastolic blood pressure; G: guideline; HDL: high density; HR: heart rate; I: intensive; LDL: low density; SBP: systolic blood pressure; TC: total cholesterol; TG: triglycerides. (DOCX) [file pone.0164608.s008.docx]

| Outcome | Group | Baseline | Month 1 | Month 2 | Month 3 | Month 6 | Change | Mean difference  (95% CI) | 2p |
| --- | --- | --- | --- | --- | --- | --- | --- | --- | --- |
| SBP | I | 147.1 (20.1) | 137.1 (17.4) | 131.8 (15.9) | 129.1 (14.6) | 130.0 (15.0) | -17.1 (15.7) | **-10.6 (-16.3, -4.8)** | **<0.001** |
|  | G | 147.0 (16.7) | - | - | - | 140.5 (15.4) | -6.5 (16.3) |  |  |
| DBP | I | 82.9 (12.1) | 77.0 (10.3) | 74.2 (8.6) | 74.1 (9.2) | 72.9 (9.6) | -10.0 (8.9) | **-5.5 (-9.1, -1.8)** | **0.004** |
|  | G | 81.2 (10.4) | - | - | - | 77.4 (11.1) | -3.9 (9.3) |  |  |
| HR | I | 70.4 (14.8) | 72.7 (14.6) | 69.6 (12.6) | 69.7 (12.3) | 69.3 (12.0) | -1.1 (12.9) | **-5.0 (-9.5, -0.4)** | **0.034** |
|  | G | 71.3 (13.9) | - | - | - | 74.8 (15.2) | 3.6 (9.2) |  |  |
| TC | I | 3.99 (0.67) | - | - | 3.89 (0.76) | 3.57 (0.76) | -0.42 (0.58) | **-0.54 (-0.90, -0.19)** | **0.003** |
|  | G | 3.88 (1.02) | - | - | - | 4.02 (1.27) | 0.13 (0.87) |  |  |
| TG | I | 1.41 (0.66) | - | - | 1.38 (0.81) | 1.33 (0.73) | -0.08 (0.5) | 0.00 (-0.22, 0.21) | 0.97 |
|  | G | 1.26 (0.57) | - | - | - | 1.21 (0.59) | -0.05 (0.37) |  |  |
| LDL-c | I | 2.02 (0.55) | - | - | 1.89 (0.58) | 1.67 (0.61) | -0.34 (0.48) | **-0.44 (-0.72, -0.15)** | **0.003** |
|  | G | 1.96 (0.69) | - | - | - | 2.06 (0.98) | 0.1 (0.67) |  |  |
| HDL-c | I | 1.35 (0.36) | - | - | 1.39 (0.42) | 1.31 (0.37) | -0.04 (0.19) | -0.12 (-0.29, 0.04) | 0.13 |
|  | G | 1.46 (0.59) | - | - | - | 1.53 (0.65) | 0.07 (0.44) |  |  |
| Non-HDL | I | 2.63 (0.6) | - | - | 2.5 (0.8) | 2.25 (0.81) | -0.38 (0.61) | **-0.44 (-0.82, -0.06)** | **0.024** |
|  | G | 2.42 (0.98) | - | - | - | 2.49 (1.3) | 0.07 (0.9) |  |  |
