# Supplementary material for: Intensive versus Guideline Blood Pressure and Lipid Lowering in Patients with Previous Stroke: Main Results from the Pilot ‘Prevention of Decline in Cognition after Stroke Trial’ (PODCAST) Randomised Controlled Trial
Source: PLoS One. 2017 Jan 17;12(1):e0164608. doi: 10.1371/journal.pone.0164608 (PMC5240987; doi:10.1371/journal.pone.0164608)
Supplement: S5 Table — Data are mean (standard deviation); comparison by ANCOVA with mean difference on treatment adjusted for baseline. (DOCX) [file pone.0164608.s009.docx]

| Month |  | Baseline | On-treatment | Mean difference | 2p |
| --- | --- | --- | --- | --- | --- |
| Systolic BP (mmHg) | Intensive | 127.5 (12.7) | 118.5 (9.3) | -10.0 (-21.2, 1.3) | 0.079 |
|  | Guideline | 127.3 (15.9) | 128.3 (14) | - | - |
| Diastolic BP (mmHg) | Intensive | 77.8 (8.4) | 69.8 (4.4) | **-6.3 (-12.2, -0.3)** | **0.041** |
|  | Guideline | 73.7 (9.2) | 73.9 (7.9) | - | - |
| Heart rate (bpm) | Intensive | 75.3 (14.4) | 71.2 (11.5) | -3.0 (-8.6, 2.6) | 0.27 |
|  | Guideline | 75.6 (12.1) | 74.3 (9.1) | - | - |
