# Supplementary material for: Intensive versus Guideline Blood Pressure and Lipid Lowering in Patients with Previous Stroke: Main Results from the Pilot ‘Prevention of Decline in Cognition after Stroke Trial’ (PODCAST) Randomised Controlled Trial
Source: PLoS One. 2017 Jan 17;12(1):e0164608. doi: 10.1371/journal.pone.0164608 (PMC5240987; doi:10.1371/journal.pone.0164608)
Supplement: S6 Table — (DOCX) [file pone.0164608.s010.docx]

| **Issue** | **Explanation** | **Result/response** |
| --- | --- | --- |
| Failure to achieve intensive systolic BP target (<125 mmHg) | Number of BP drugs (or increases in drug dosing) not escalated in 17 (45.9%) patients randomised into the intensive arm | Repeat messages to investigators to intensify BP-lowering treatment |
| Failure to achieve difference of 1.0 mmol/l LDL-c between intensive and guideline groups | Third generation statins used in guideline lipid group, reflecting guideline drift. | Repeat messages sent to investigators to use guideline lipid-lowering treatment |
| Failure to achieve intensive LDL-cholesterol target (<1.3 mmol/l) | Third generation statins (atorvastatin, rosuvastatin), with or without addition of ezetimibe, not used in patients randomised into the intensive arm. | Repeat messages sent to investigators to intensify lipid-lowering treatment |
|  | Limited availability of cholestyramine |  |
| Development of dementia at lower rate than expected across the trial. | Unclear, since patients nominally at risk of developing dementia post stroke and with non-normal baseline cognition scores. | Reduced statistical power to detect treatment effects on development of dementia |
