# Supplementary material for: Intensive versus Guideline Blood Pressure and Lipid Lowering in Patients with Previous Stroke: Main Results from the Pilot ‘Prevention of Decline in Cognition after Stroke Trial’ (PODCAST) Randomised Controlled Trial
Source: PLoS One. 2017 Jan 17;12(1):e0164608. doi: 10.1371/journal.pone.0164608 (PMC5240987; doi:10.1371/journal.pone.0164608)
Supplement: S7 Table — Data are mean (standard deviation). Comparisons by t-test with Bonferroni adjustment for multiple comparisons. (DOCX) [file pone.0164608.s011.docx]

| Group |  | Month 0 | Month 6 | Month 18 | Month 30 |
| --- | --- | --- | --- | --- | --- |
| BP | Patients | 41/42 | 38/39 | 33/32 | 13/11 |
|  | Intensive | 85.7 (8.1) | 81.6 (18.0) | 85.2(20.9) | 84.0 (26.2) |
|  | Guideline | 86.5 (7.4) | 87.5 (7.4) | 87.0 (8.8) | 83.8 (8.6) |
|  | Difference | - | -5.9 (-12.2, 0.3) | -1.7 (-9.7, 6.3) | 0.2 (-17.0, 17.3) |
|  | 2p | - | 0.18 | 1.00 | 1.00 |
| Lipids | Patients | 39/38 | 37/35 | 29/30 | 11/11 |
|  | Intensive | 87.6 (7.7) | 88.8 (7.2) | 89.8 (8.6) | 88.9 (9.2) |
|  | Guideline | 84.4 (7.3) | 80.3 (18.1) | 82.1 (21.3) | 79.8 (27.6) |
|  | Difference | - | 8.4 (2.0, 14.9) | 7.7 (-0.8, 16.2) | 9.1 (-9.2, 27.4) |
|  | 2p | - | **0.032** | 0.22 | 0.94 |
