# Supplementary material for: Intensive versus Guideline Blood Pressure and Lipid Lowering in Patients with Previous Stroke: Main Results from the Pilot ‘Prevention of Decline in Cognition after Stroke Trial’ (PODCAST) Randomised Controlled Trial
Source: PLoS One. 2017 Jan 17;12(1):e0164608. doi: 10.1371/journal.pone.0164608 (PMC5240987; doi:10.1371/journal.pone.0164608)
Supplement: S8 Table — Data are number (%). Comparison by Chi-square test. † p<0.05. (DOCX) [file pone.0164608.s012.docx]

|  | BP |  | Lipid |  |
| --- | --- | --- | --- | --- |
| Cause | Intensive | Guideline | Intensive | Guideline |
| Cardiovascular | 5 (12.2) | 6 (14.3) | 3 (7.7) | 7 (18.4) |
| Atrial fibrillation | 2 (4.9) | 1 (2.4) | 0 (0) | 3 (7.9) |
| Sudden cardiac death | 0 (0) | 1 (2.4) | 0 (0) | 1 (2.6) |
| Genitourinary | 4 (9.8) | 2 (4.8) | 2 (5.1) | 4 (10.5) |
| Urinary tract infection | 3 (7.3) | 1 (2.4) | 1 (2.6) | 3 (7.9) |
| Gastrointestinal | 2 (4.9) | 1 (2.4) | 0 (0) | 3 (7.9) |
| Haematological | 0 (0) | 2 (4.8) | 1 (2.6) | 1 (2.6) |
| Metabolic/Endocrine | 1 (2.4) | 0 (0) | 0 (0) | 1 (2.6) |
| Musculoskeletal | 1 (2.4) | 2 (4.8) | 3 (7.7) | 0 (0) |
| Nervous system | 3 (7.3) | 5 (11.9) | 3 (7.7) | 3 (7.9) |
| Respiratory | 5 (12.2) | 4 (9.5) | 2 (5.1) | 7 (18.4) |
| Chest infection or pneumonia | 4 (9.8) | 3 (7.1) | **1 (2.6) †** | **6 (15.8)** |
| Miscellaneous | 14 (34.2) | 11 (26.2) | 11 (28.2) | 14 (36.8) |
| Fall | 7 (17.1) | 4 (9.5) | 4 (10.3) | 7 (18.4) |
| Malignancy | 2 (4.8) | 1 (2.4) | 1 (2.6) | 2 (5.3) |
| Total | 18 (43.9) | 22 (52.4) | 17 (43.6) | 21 (55.3) |
