# Supplementary material for: Intensive versus Guideline Blood Pressure and Lipid Lowering in Patients with Previous Stroke: Main Results from the Pilot ‘Prevention of Decline in Cognition after Stroke Trial’ (PODCAST) Randomised Controlled Trial
Source: PLoS One. 2017 Jan 17;12(1):e0164608. doi: 10.1371/journal.pone.0164608 (PMC5240987; doi:10.1371/journal.pone.0164608)
Supplement: S9 Table — Data are number. (DOCX) [file pone.0164608.s013.docx]

| Cause of death | BP |  | Lipid |  |
| --- | --- | --- | --- | --- |
|  | Intensive | Guideline | Intensive | Guideline |
| Dementia | 1 | 0 | 0 | 1 |
| Stroke | 1 | 0 | 1 | 0 |
| Sudden cardiac death | 0 | 1 | 0 | 1 |
| Death unattended | 0 | 1 | 1 | 0 |
| Tumour - Malignant | 1 | 1 | 1 | 1 |
| Pneumonia | 1 | 0 | 0 | 1 |
| Total | 4 | 3 | 3 | 4 |
