# Supplementary material for: Intensive versus Guideline Blood Pressure and Lipid Lowering in Patients with Previous Stroke: Main Results from the Pilot ‘Prevention of Decline in Cognition after Stroke Trial’ (PODCAST) Randomised Controlled Trial
Source: PLoS One. 2017 Jan 17;12(1):e0164608. doi: 10.1371/journal.pone.0164608 (PMC5240987; doi:10.1371/journal.pone.0164608)
Supplement: S10 Table — Data are number of events. (DOCX) [file pone.0164608.s014.docx]

| Event |  |  |  |  | BP |  | Lipid |  |
| --- | --- | --- | --- | --- | --- | --- | --- | --- |
|  |  |  |  |  | Intensive | Guideline | Intensive | Guideline |
| N |  |  |  |  | 41 | 42 | 39 | 38 |
| Dementia |  |  |  |  |  |  |  |  |
| All (%) |  |  |  |  | 2 (4.9) | 0 (0) | 0 (0) | 2 (5.3) |
| Vascular |  |  |  |  | 2 (4.9) | 0 (0) | 0 (0) | 2 (5.3) |
| Mixed |  |  |  |  | 0 (0) | 0 (0) | 0 (0) | 0 (0) |
| Vascular |  |  |  |  |  |  |  |  |
| Stroke recurrence |  |  |  |  |  |  |  |  |
| Ischaemic |  |  |  |  | 0 (0) | 2 (4.8) | 1 (2.6) | 1 (2.6) |
| Haemorrhagic |  |  |  |  | 1 (2.4) | 1 (2.4) | 1 (2.6) | 0 |
| Fatal |  |  |  |  | 1 (2.4) | 0 (0) | 1 (2.6) | 0 |
| Non-fatal |  |  |  |  | 0 (0) | 3 (7.1) | 1 (2.6) | 1 (2.6) |
| MI |  |  |  |  | 0 (0) | 1 (2.4) | 1 (2.6) | 0 |
| Vascular composite |  |  |  |  | 1 (2.4) | 4 (9.5) | 3 (7.7) | 1 (2.6) |
