# Supplementary material for: Intensive versus Guideline Blood Pressure and Lipid Lowering in Patients with Previous Stroke: Main Results from the Pilot ‘Prevention of Decline in Cognition after Stroke Trial’ (PODCAST) Randomised Controlled Trial
Source: PLoS One. 2017 Jan 17;12(1):e0164608. doi: 10.1371/journal.pone.0164608 (PMC5240987; doi:10.1371/journal.pone.0164608)
Supplement: S11 Table — (DOCX) [file pone.0164608.s015.docx]

| **Trial no.** | **Theme and comment** |
| --- | --- |
|  | **I’ve enjoyed it…** |
| 03 | I've enjoyed it along the way |
| 19 | I've enjoyed it. I've got to know you all. I will miss it |
| 74 | I've enjoyed it! |
|  | **I hope it helps others…** |
| 15 | I hope it helps others as it has certainly helped me |
| 62 | I just hope it does someone else good |
|  | **It’s a shame it had to come to an end…** |
| 22 | I felt very much looked after, it's sad to see it end |
| 27 | Sad to see it come to an end, I did feel very well looked after |
| 41 | I am disappointed to see the trial having to end. It's been beneficial to me. Best of luck with the results from the wife and I |
| 47 | It's very sad to see the trial ending early. It worked well for me; I've benefitted from it. I hope the results will help others |
| 48 | I'm so sad this has to end. I felt I was getting looked after well, any moans I had I felt comfortable to tell them, they were so kind and patient with me. I felt reassured visiting them |
| 72 | I did it for my own benefit but I also hope it helps others. It's a great shame that the trial had to finish before it ran its course |
| 81 | Its been a pleasure. I hope the information gathered proves useful. It’s a shame it had to end |
|  | **I felt very well looked after…** |
| 17 | I felt very much looked after" "it was reassuring to know I was being so closely monitored" - "I hope I will be okay on my own - let's hope so |
| 21 | The trial has been really helpful to me these past few years. I felt very well looked after. The team at the hospital were fantastic. Thank you to all at PODCAST |
| 34 | Thank you, I felt well looked after |
|  | **Thank you…** |
| 46 | You've really helped me, thank you so much |
| 58 | Thank you and I hope it has done some good? |
| 64 | It was a positive experience for myself and my wife. Taking part helped me build my confidence.  From participant’s wife - I feel this project/study was really important for my husband and other stroke patients. It really helped raise his confidence without it I feel that he would have become depressed. It gave him the confidence to go out and try other things, to keep him busy. It was really good! Thank you |
| 65 | Thank you, you have all been so fantastic! Last week at my final hospital visit the team showed me around the new stroke unit, which was marvellous to see and very kind of them to share with me. Brilliant - I can't praise all of you enough! I have been extremely well looked after |
| 66 | It's been a pleasure! Thank you to all. I am due to be signed up to another clinical trial *(a lipids trial)*, just have to wait 30 days. It keeps me busy! |
| 67 | It's had its benefits - because of this my nephew has taken part in several 'Mud-runs' to raise money for Stroke charities. Thank you |
| 73 | Thank you for being there for us |
